# A Proof of Concept Combined Using Mixed Reality for Personalized Neurorehabilitation of Cerebellar Ataxic Patients

**DOI:** 10.3390/s23031680

**Published:** 2023-02-03

**Authors:** Michela Franzò, Andrada Pica, Simona Pascucci, Mariano Serrao, Franco Marinozzi, Fabiano Bini

**Affiliations:** 1 Department of Mechanical and Aerospace Engineering, “Sapienza” University of Rome, 00184 Rome, Italy; 2 National Centre for Clinical Excellence, Healthcare Quality and Safety, Italian National Institute of Health, 00161 Rome, Italy; 3 Department of Medical and Surgical Sciences and Biotechnologies, “Sapienza” University of Rome, 00185 Rome, Italy

**Keywords:** motion capture, inertial measurement unit, health monitoring, mixed reality, rehabilitation engineering, ataxia, exergaming

## Abstract

Background: Guidelines for degenerative cerebellar ataxia neurorehabilitation suggest intensive coordinative training based on physiotherapeutic exercises. Scientific studies demonstrate virtual exergaming therapeutic value. However, patient-based personalization, post processing analyses and specific audio-visual feedbacks are not provided. This paper presents a wearable motion tracking system with recording and playback features. This system has been specifically designed for ataxic patients, for upper limbs coordination studies with the aim to retrain movement in a neurorehabilitation setting. Suggestions from neurologists and ataxia patients were considered to overcome the shortcomings of virtual systems and implement exergaming. Methods: The system consists of the mixed-reality headset Hololens2 and a proprietary exergaming implemented in Unity. Hololens2 can track and save upper limb parameters, head position and gaze direction in runtime. Results: Data collected from a healthy subject are reported to demonstrate features and outputs of the system. Conclusions: Although further improvements and validations are needed, the system meets the needs of a dynamic patient-based exergaming for patients with cerebellar ataxia. Compared with existing solutions, the mixed-reality system is designed to provide an effective and safe therapeutic exergaming that supports both primary and secondary goals of an exergaming: what a patient should do and how patient actions should be performed.

## 1. Introduction

Rehabilitation is a vast field of the health system and research. Technological advances participate in the steady growth of the rehabilitation field, providing efficient and innovative solutions. The overall span of technology involved in rehabilitation protocols is extremely wide: from assistive devices [1], robotics [2], the use of optoelectronic systems [3], inertial measurement units [4], virtual and augmented reality environments [5,6] for the deployment of rehabilitative therapies. Particular interest has arisen in the introduction of Virtual Reality (VR) and Augmented Reality (AR) devices that today are most studied for their applicability in rehabilitation. Knowing the position of the patient in real time allows for realistic interaction rather than passive participation [7], and makes post-processing analysis available. Different studies [8,9,10] have validated the capacity of these devices to support rehabilitative activities, generating biomechanical outcomes comparable to gold standard outcomes. In these studies, the performance of Virtual and Augmented Reality devices is compared to gait analysis and motion systems globally considered as references for tracking motion, such as Vicon and MoCap.

In particular, the headset Microsoft HoloLens (HL) and its second version (HL2) have introduced a new application of AR technology, by providing interactive digital stimuli in the real-life context, starting, in this manner, in the Mixed Reality (MR) era. HL2 has the potential to be the next generation of wearable technology assistance for patients who need support in daily activities, providing virtual input contextualized in the real world through objectively quantifying patient movements [8].

In this paper, a wearable motion tracking system with recording and playback features is presented. This system has been designed for upper limb coordination studies with the aim of retraining the perception and precision of movements in a rehabilitation setting. The system consists of the headset HL2 and a central computer with a proprietary code implemented in the open-source development platform Unity (v.2020.3.0f1). The presented Proof of Concept (PoC) is specifically realized for ataxic patients.

Cerebellar ataxia is a degenerative genetic disease that involves the peripheral and central nervous systems [11]. The cerebellum is the main damaged organ, and this condition causes, for ataxic patients, an absence of coherent representation of themselves within the external ambient. For instance, when performing a movement to reach an object, these patients commit errors of measurement and direction that they try to balance with other movements in the opposite direction [12,13,14,15]. Guidelines for ataxia neurorehabilitation suggest intensive coordinative training based on physiotherapeutic exercises. Therefore, for this type of pathology, some virtual games are currently presented, considering that exergaming training is demonstrated to have therapeutic value in ataxic patients [16,17]. However, compared with these solutions, which simulate already existing console virtual games [18,19], the PoC proposed in this study is dynamic, specific for neurorehabilitation, settable and patient-centered. Moreover, the MR system is designed to provide effective and safe therapeutic exergaming that supports both primary and secondary goals of exergaming: what a patient should do and how patient actions should be performed [20].

Neurologists and advanced ataxic patients were consulted during the design of the exergaming in order to respect both their needs in carrying out an efficient therapy. Principal requests of the neurologists in a rehabilitation system are the possibility of setting the game to be patient-centered and to control the patient’s performance during the training. Instead, patients’ needs are generally entertainment, comfortability and patient-friendly comprehensibility. The game settings should be set through simple graphical elements or vocal commands, and the interaction mechanisms should be intuitive. Therefore, the aim of the present project is to provide an innovative wearable-sensor system, which is specific to the cerebellar ataxia neurorehabilitation, providing features beneficial for therapeutic progresses, entertainment for greater patient concentration, research results and future development in daily assistance technology.

## 2. Related Work

Several studies have been published in the last decade that have examined the efficacy of the innovative technologies in rehabilitation. Special interest has been aimed at the introduction of Virtual (VR) and Mixed Reality (MR) in rehabilitation guidelines.

Feng and co-workers [21] demonstrated the advantage of using VR for rehabilitation purposes because of the different types of feedback that virtual games can provide. Real-time feedback improves the patient’s cognitive sensation, increases interest in the exergaming and motivates repetition. Rehabilitation is based on the repetition of an action and many trial results have shown that patients can learn the skill and apply it to the real world.

Actually, in ataxia neurorehabilitation, several studies [18,19] have demonstrated the effectiveness of high-intensity coordinative training in leading to a significant benefit in patients with degenerative ataxia. Even patients with advanced neurodegeneration conditions could benefit from a training program based on 2D whole-body controlled videogames. One of the latest examples of 3D virtual games on ataxic patients is the study of Ayvat and colleagues [16]. In this research [16], the results showed improvement in balance, stability limits and cognitive processing, with a consequential general reduction in ataxia severity. Moreover, studies like [22] combined exergaming and sensors for motion analysis.

VR and devices with head-mounted displays (HMD) were the first to be invented and introduced in the medical field. Consequently, most studies available in the literature have considered VR devices for rehabilitation. MR was introduced with the invention of holographic devices. HL2 is the latest version of the Microsoft holographic device for MR. An in-depth literature review of the various applications of HL2 since it was invented in 2017 was carried out by Park and co-workers [15]. The principal categories of application are medical and surgical aids and systems, medical education and simulation, industrial engineering, architecture and civil engineering. Currently, most applications registered in the literature concern the medical field and, through the variety of functions provided to the neurologists, the interaction in a total virtual world is the most adopted. Instead, [23] presented a systematic review which reported the impact of MR and hybrid approaches (virtual and physical at the same time) on medical simulation. In some studies [23,24], the combination of MR and hybrid approaches has been favored.

HL2 allows for the acquisition of head position and rotation with an inertial measurement unit (IMU) integrated into the headset, gaze direction by means of an algorithm of eye tracking, hand position and gestures, through a body-tracking algorithm.

In [7], the IMU of HL2 was compared with a gold standard reference sensor; 0.87 percent was the highest deviation from the starting position and a final position after the traveled path of 5655 m. Instead, in [8], the IMU was compared to other reference systems of gait acquisition. In static acquisition, HL2 measures were statistically equivalent (error ≤ 5%, *p* < 0.05, 95% confidence level) to the MoCap Technology measures, whereas its reliability in walking trials was found to be excellent, with intra-class correlation coefficient (ICC(2,1)) values ≥ 0.98.

According to [25], one of the last and most specific studies on HL2 body tracking, the recognition and tracking of patient hands still has room for improvement. Indeed, circumstances during tracking, such as rotation of the head, position of the hand inside the field of view, frequencies of acquisition and dimension of the hand, influence accuracy and repeatability, albeit in negligible amounts in some fields of application.

The eye tracking algorithm of HL2 also needs some improvements. The information regarding accuracy reported by the manufacturer is vague and not always respected. The study presented in [26] demonstrated that the spatial accuracy results by original data acquisitions are consistently worse than those reported by the manufacturer, even though a recalibration of the eye sensor improves it. In the study [27], spatial accuracy was observed in different conditions. While the subject was sitting, evaluation of the spatial accuracy was better than the one registered while walking.

The project presented here is an upgrade of a previous prototype implemented on the LabView platform with the Microsoft Kinect v2 [28,29]. In the previous system, bidimensional VR exergaming was designed specifically for ataxic patients, following neurologist and patient suggestions. In these phases, exergaming design and parameters for patient-personalization were perfected. Then, a first approach to the HL2 was performed, reproducing the project in the MR environment [30]. Consequently, the project was re-designed and assessed for MR, and the current system was implemented.

## 3. Materials and Methods

### 3.1. The PoC Design

**Figure 1:**
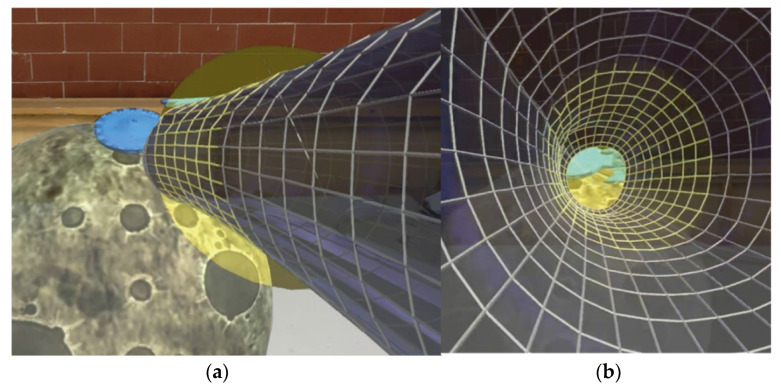
Frames of the HL2 view during the exergaming: (**a**) space environment considered for the exergaming from a lateral vision, in blue the spaceship the patient has to reach; (**b**) space environment from a frontal vision, inside the wormhole-like object that limits the volume of work.

The exergaming is designed to be played in a sitting position. The implemented exercises involve pointing, where the subject has to reach a target with the hand and to eventually follow its movements in the 3D space around him. The virtual location chosen for exergaming is space, the target to reach is a spaceship on a planet and the audio feedback is typical science fiction sounds (Figure 1).

In exergaming 1, the patient has to reach the spaceship; grab it; move it to a new planet that appears near them in a different position. The subject has to repeat this activity for the following planets. In exergaming 2, first, the patient has to grab the spaceship. Then, the spaceship starts moving following a square trajectory on the frontal plane. The distance of the frontal plane is settable in Unity. Finally, the patient has to reproduce the same trajectory by following the spaceship with their hand. To better converge the gaze of the subject and to control the position of the hand during both exergaming models, a limitation of the volume of work is displayed. A 3D wormhole-like object, similar to a truncated cone that is hollow inside, is shown in front of the subject with its smallest extremity centered on the target and its largest on the shoulder of the subject. The subject has to reach the target without exiting the wormhole. The best trajectory possible is the central axis of the wormhole.

### 3.2. Hardware Design

The system is composed of HL2 and a central computer with an exergaming implemented in Unity. The version of Unity used is 2020.3.30f1 and the MRTK 2.7 Microsoft-driven project was installed to provide components and features used to develop a cross-platform MR project. The system uses the Microsoft-Unity Holographic Remoting function provided by the XR asset. In Holographic Remoting settings, the project runs on the computer instead of on HL2; therefore, the holographic device is connected via USB-C port to the computer and its input/output are managed by the Unity software. The computer is a Windows 10 64-bit device with 32 GB RAM, Intel i7-1050H CPU 2.60GHz and an Internal Intel Video BIOS UHD graphic.

The model of the spaceship and the feedback sounds are free and available online, while the wormhole was created on the open-source computer graphics software Blender and customized to match the neurologists’ requests.

### 3.3. Software Design

The software is implemented in Unity to build the 3D virtual game specifically for Microsoft Platforms. Several C# scripts were requested to design the exergaming comprehensive of all features (feedbacks, animations, vocal commands, etc.). The software manages the HL2, providing information about the 3D game objects and feedbacks to be shown to the patient while receiving data acquisitions from the sensor. Moreover, the software provides an interface for the patient to see the view through the HL2 in real time on the monitor, interact with the 3D objects and choose the game settings. The patient interface is intended to be managed by the clinician, who prepares the rehabilitation training.

**Figure 2:**
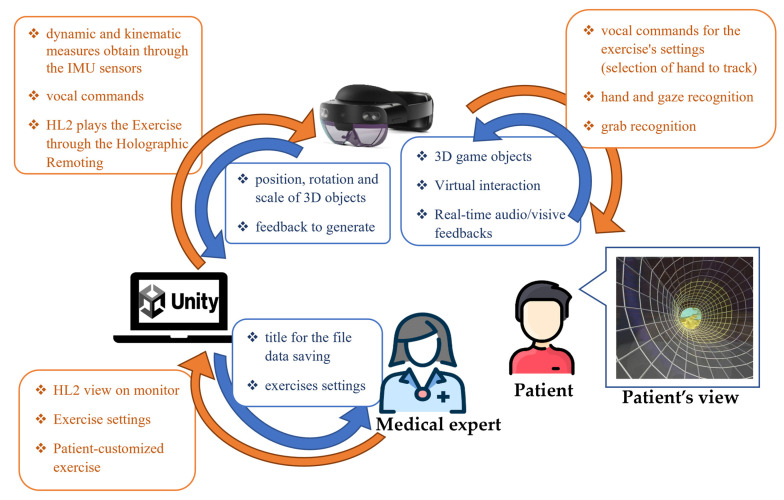
Scheme of input/output across the PoC: HL2, computer, medical expert interface interactive for clinician and the patient that wears HL2.

Overall, the PoC is included in an interconnected structure involving two actors: the patient and the medical expert. The patient wears the HL2 and performs the training, while the medical expert is the clinician that interacts with the computer and selects the game settings. Devices and actors exchange information and commands (Figure 2).

The 3D game object positions are pre-set by the software. However, the medical expert can change the position in real time according to his/her necessities, such as bringing the target nearer, matching the length of the patient’s arm. The HL2 tracks the hand movements in space and a positive feedback is generated when the hand of the subject is inside the volume-limitation, i.e., the wormhole, and a negative feedback when outside. The two exergaming modes are implemented to be performed with both hands. All 3D game objects change their position and rotation according to the hand selected, allowing the patient to comfortably perform the exergaming. Consequently, the wormhole must always be accordingly centered on the left or right shoulder.

Through the Unity interface, the neurologist can first choose the exergaming to be performed and the hand that will be used. Game settings can be selected. The available game settings are the number of times the exergaming has to be repeated by the patient and the difficulty of the exergaming. Difficulty is associated with the volume of limitation, i.e., the larger the volume of limitations, the simpler the exergaming. Therefore, the medical expert can change the scale of the wormhole to make it smaller or larger.

The HL2 can also recognize some gestures of the hands. The gesture used in these applications is the “grab”; i.e., pressing the index finger and the thumb together. The target is considered reached when the HL2 registers a “grab” action on the target. The “grab” action on the target starts the feedback according to the exergaming that is running. Specifically, in exergaming 1, when the target is “grabbed”, a sound is generated and the target and all other 3D objects of the game change their position in the successive one. Then, the action of reach and “grab” can be repeated with the target in the new position. In exergaming 2, the “grab” of the target starts the animation of the 3D objects of the game. The wormhole starts rotating and moving, following a specific track on a frontal plane. The subject has to follow the same track, leaving the hand inside the wormhole in order to obtain positive audio-visual feedbacks.

The possibility of using vocal commands for the selection of the exergaming and the hand to track in the performance is provided. The vocal commands are registered and interpreted by the HL2 and elaborated by the Unity project.

**Figure 3:**
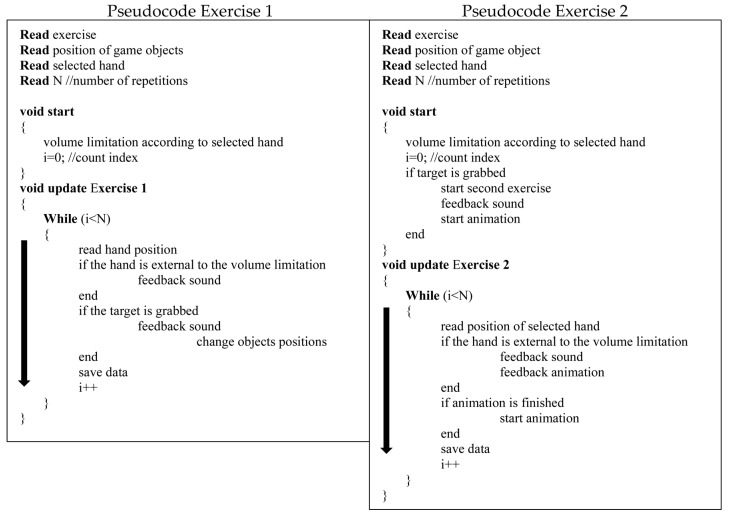
Pseudocodes of the exergaming implemented in C# style and reporting the conditions and feedbacks generated.

Figure 3 describes the steps followed by the Unity project to implement both exergaming exercises.

### 3.4. Data Acquired

The system records information in text files (.txt) during the exergaming. The information collected are both data acquired by the HL2 and output to evaluate the patient performance. The HL2 acquires the kinematic and dynamic measurements of both hands relative to the head system of reference:

− Position coordinates (meters)
− Euler rotations (degrees)
− Velocity (m/s)

Moreover, the IMU inside the HL2 acquires position coordinates and Euler rotations of the head, while the gaze position (meters) and Euler rotations are saved by the eye-tracking algorithm. The information collected on the performance is:

− Current positions and rotations of all 3D game objects
− Time instant of recognition of a “grab” gesture on the target and time instant of releasing the grab
− Time instant of exit or enter the volume of limitation of the hands

With this information, the post-processing reconstruction and evaluation of the performance is possible. All data are registered by different C# scripts running at the same time. The data are registered in reference to the same time line that starts with the beginning of the exergaming. The acquisition frequency of the HL2 parameters is not set. The frequency changes according to the ability of the device in the single instant to elaborate and acquire data.

### 3.5. Collected Data Analysis

A healthy, 30-year-old right-handed subject tried the PoC.

The data collected from the healthy subject were reported to show features and outputs of the PoC. The two exergaming exercises were performed twice with both hands. The wormhole scale was set to the default values. The diameter of the smallest extremity was set to 7 cm, while the largest was set to 70 cm. Therefore, given the large volume of limitations, the exergaming was characterized by low difficulty.

## 4. Results

**Figure 4:**
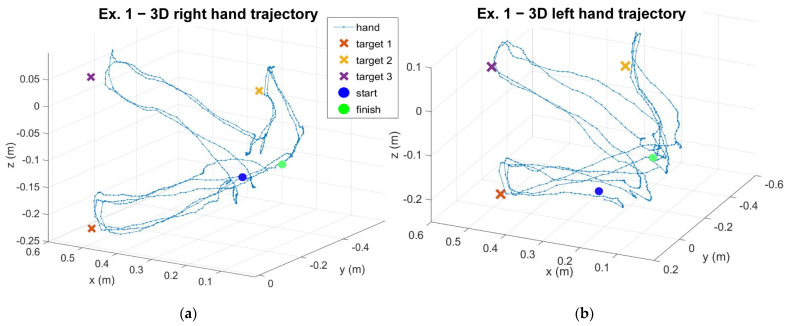

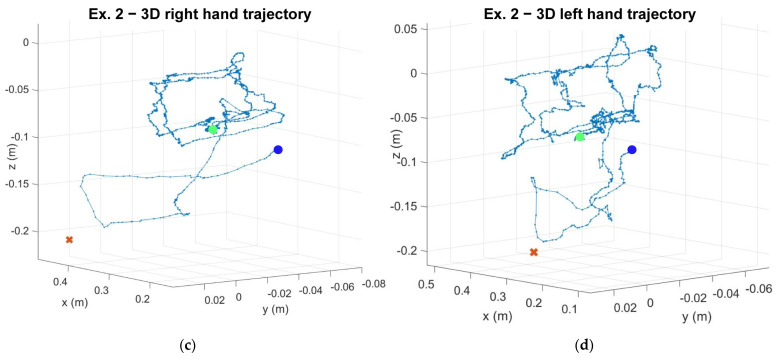
Graphs of the trajectories of the subject’s hand: (**a**) trajectory of the right hand during exergaming 1, (**b**) trajectory of the left hand during exergaming 1, (**c**) trajectory of the right hand during exergaming 2, (**d**) trajectory of the left hand during exergaming 2. The cross represents the position of the targets that are “grabbed” by the subject in the exergaming.

Figure 4 reports the trajectories of the right and left hands for both exergaming exercises. Each exergaming was consecutively performed two times, so the data acquired corresponded to both repetitions together. The targets are represented by cross symbols on the graphs. In the graphs reporting exergaming 1 trajectories (Figure 4a,b), the positions of the three targets are reported. In exergaming 2 (Figure 4c,d), the movement of the target is recognizable; a rectangular trajectory on the frontal plane (i.e., yz plane) of approximately 60 cm on the horizontal side and 3 cm on the vertical side.

Figures S1–S8 report the kinematic quantities of both hands during the two exergaming exercises. Figure 5 shows the 3D position of the IMU sensor integrated into the device during the acquisitions, while Figures S9–S12 report the rotation measured for the head.

**Figure 6:**
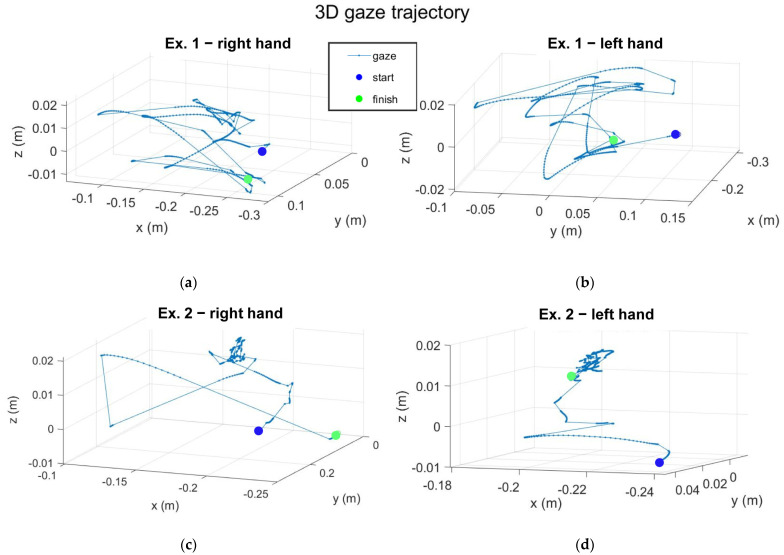
Graphs of the trajectories of the subject’s gaze: (**a**) trajectory during exergaming 1 performed with the right hand, (**b**) trajectory during exergaming 1 performed with the left hand, (**c**) trajectory during exergaming 2 performed with the right hand, (**d**) trajectory during exergaming 2 performed with the left hand.

Figure 6 shows the 3D trajectories of the gaze registered by the device during the acquisitions, while the gaze rotations are reported in Figures S13–S16.

In Table 1, we report the information concerning the acquisitions. In particular, the total time of the acquisitions, the number of samples acquired and the number of times the feedback was generated.

The feedback registered regards the exit of the hand from the volume of limitation (wormhole). The acquisition time of exergaming 2 is implicitly set, since the subject has to follow the animation of the game-objects already implemented. The small difference among acquisitions is caused by the time the subject spent reaching and grabbing the target in order to start the animation.

## 5. Discussion

The PoC was designed to be used following a specific procedure of acquisition, which provides a guide on how to connect and set the HL2 for Holographic Remoting, and information on the patient interface to manage the system.

The Holographic Remoting functionality provides a patient interface in real time to the clinician who can follow the training step-by-step. Moreover, this functionality avoids burdening the HL2 RAM, for which the amount of operation to perform was excessive despite the settings of the project being for MR game development. However, the patient was limited in his movements around the room because of the USB cable that connected the HL2 to the computer. Actually, this inconvenience was negligible considering that the majority of ataxic patients have problems standing and use wheelchairs. According to this necessity of ataxic patients, the exergaming was structured for upper limb rehabilitation and it was designed to be played from a sitting position.

The results taken from a healthy subject showed the general outputs of the project and the outcomes for future analysis on the quality of the experience. Analysis of the kinematic quantities showed the presence of pathology (Figure S1 to Figure S8). Ataxic patients tried to leave the hand in the same orientation, without rotating it (especially for the Yaw angle), and they made effort to control their movement, so velocity and acceleration should have had a roughly constant value, except for instants of rapid changes of direction. The changes in direction occur when the patient is in an advanced ataxic-phase or when their concentration drops. Accordingly, the trajectories in space (Figure 4) of an ataxic patient should be more confused than a healthy subject, and with rapid changes in direction. Moreover, trajectory analysis can also basically show the dominant hand of the subject. In Figure 4c,d, the trajectory designed by the right-handed subject with the left hand was more confused in comparison to the trajectory of the right hand. The position of the target and the wormhole were also saved, and the targets position is shown on the graphs. The time instant of the “grab” action was tracked, as well as the instant in which the sound feedback was generated. This implementation allows us to know the total amount of feedback and the number of times the subject hand exited from the wormhole. The latter is important to quantify the impact of the exergaming on the patient in a long-run therapy, and with different difficulty settings. In fact, a high number of feedback signals is indicative of noticeable difficulties of the subject in performing exergaming. In this case, the subject has to make efforts to achieve a better performance. In this context, as seen from the data reported in Table 1, the right-handed healthy subject generated more feedback with the left hand than with the right hand. In Figures S9–S12, the rotation of the head of a healthy subject was approximately null, but the patient was expected to have a high rotation of the head because of the difficulties in keeping the same posture. Signals of the gaze direction (Figures S13–S16) showed a null roll angle of rotation because of the degree of freedom of the iris.

The vocal commands were coherent and intuitive. They were properly introduced to reduce the possibility of the subject autonomously selecting the game settings without using the hand to press a button. For the ultimate purpose of ataxic patient rehabilitation, the introduction of vocal commands was fundamental. Moreover, the exergaming was designed to engage and entertain the subject, and, in addition, to boost the need to increase their concentration in order to perform the exergaming. These factors are fundamental in rehabilitation.

In order to improve the hand recognition of the HL2 and to ensure a clearer experience, it is necessary to provide an environment with low direct illumination and to prevent other subjects from passing through the device’s field of view, so that no hands other than those of the wearer are tracked. In future studies, statistically significant groups of control and groups of ataxic patients will be formed to perform statistical analysis and evaluate the prototype.

Further analyses of errors during the exergaming performance will be performed in the framework of patients’ acquisitions in order to obtain a detailed evaluation of patient performance. According to studies [7,8,25,26,27], the HL2 device is already validated for tracking the joint algorithm in similar conditions in healthy subjects.

The prototype considers the generic needs of patients with ataxia. Different ataxia rating scales or scores have been developed to evaluate and classify the disorder [31]. On these scales, different symptoms are evaluated and the severity is rated. Ataxic patients develop, in the pathology progression, different cognitive and motor symptoms [32,33]. Consequently, ataxic patients could find specific and different difficulties in performing and approaching exergaming. Therefore, the proposed prototype may not be suitable for all patients and additional requirements will have to be taken into consideration. The PoC can be integrated into rehabilitation treatment for a group of patients for which data acquired are compared with a control group of healthy subjects [28,29,30]. Furthermore, with the PoC, it is possible to apply the specific clinical assessment of ataxia using the SARA (Scale for the Assessment and Rating of Ataxia) scale and GOAL (Goal Attainment Score) score [18].

The PoC was designed in collaboration with the consensus of neurologists and ataxic patients, because their opinions are relevant for its correct functioning. In particular, the presence of neurologists is crucial for cognitive, physical and psychological support to the patient during the exergaming, but also the patient has to be collaborative during the support to avoid problems arising. This is a preliminary study to better investigate how the device could be used in the framework of neurorehabilitation. It will be necessary to modify the exergaming by introducing exergaming nearer to occupational therapy, in which the 3D object will be a realistic object, such as a tea cup, a pen or a fork, in accordance with the specific guidelines.

After validation with a group of patients following a specific neurorehabilitation protocol, it could define new guidance.

Currently, the system is not yet ready to be used as a home-care device and the therapists need to be trained in its use.

To support the innovative contribution of the system presented related to upper limb rehabilitation for degenerative ataxic patients, a comparison of the systems present in the literature is reported in Table 2.

## 6. Conclusions

Technological progress has led to more sophisticated sensors [9,36,37,38], devices [8,28] and methodologies [18,21,30] to investigate pathological conditions and to provide improvements in rehabilitation outcomes. The PoC presented in this study is a functional proposal for rehabilitation purposes, with the use of HL2 as a body movement sensor for patients with neurological pathology. The PoC will need subsequent software and hardware improvements. Although the headset is a commercial device, the PoC has been considered acceptable by neurologists for rehabilitative applications. In addition, the design dynamic allowed for setting patient-based exergaming. In future, it could be possible to differentiate the exergaming proposed, depending on the severity of the patient’s pathology. Virtual devices on the market and in the literature have been unsatisfactory for the needs of neurologists and ataxic patients. The PoC presented in this study is tailored to cerebellar ataxia comorbidities, and the validation process will be performed with the engagement of ataxic patients, although, in future, we do not exclude the possibility of validating its use for other diseases.

## Figures and Tables

**Figure 5:**
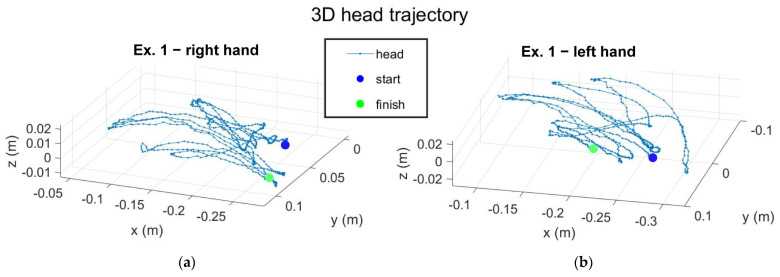

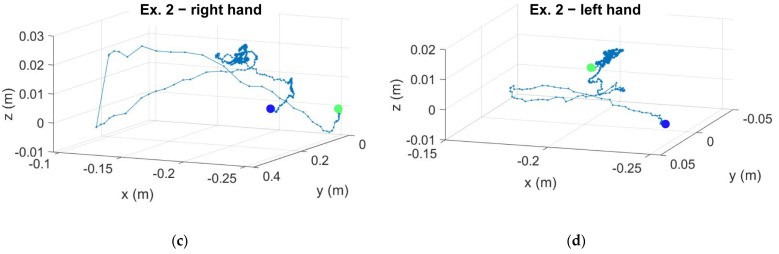
Graphs of the trajectories of the subject’s head: (**a**) trajectory during exergaming 1 performed with the right hand, (**b**) trajectory during exergaming 1 performed with the left hand, (**c**) trajectory during exergaming 2 performed with the right hand, (**d**) trajectory during exergaming 2 performed with the left hand.

**Table 1:** Data acquired by the HL2 for the two exergaming exercises. The data refer to the two repetitions consecutively performed.

Exergaming (2 Repetitions)	Hand	Acquisition Time (s)	N° Samples	N° Exit Feedback
Exergaming 1	Right	25.67	729	9
	Left	21.96	600	16
Exergaming 2	Right	44.37	1328	31
	Left	44.70	1340	52

**Table 2:** Comparison of exergaming used for upper limb rehabilitation for ataxic patients.

	Traditional	Xbox and Nintendo Wii Exergaming [16,18,19,34]	VR Headset and Controller [17]	2D VR IMU Sensors [35]	2D VR Marker-less Tracking for Ataxic Patients [28,29]	2D AR System without Headset [15]	System Proposed: 3D MR Tailored for Ataxic Patients
**PRO**	Real world; Real interaction; Human interaction; No devices; Patient-based; Free; No controller;	Entertaining; Economic; Home-Care; Dedicated or external tracking; patient-friendly; audio-visive feedback; No controller;	Patient-based; Economic; Home-Care; Data acquisition; Controller tracking; patient-friendly; audio-visive feedback; 3D virtual world;	Entertaining; IMU tracking; Data acquisition; audio-visive feedback; No controller;	Economic; Home-Care; Patient-based; Marker-less tracking; Data acquisition; patient-friendly; audio-visive feedback; No controller;	Entertaining; Home-Care; Marker-less tracking; Data acquisition; audio-visive feedback; gesture recognition; No controller; Object interaction;	Entertaining; Patient-based; Virtual object in real world; Data acquisition; gesture recognition; No controller; Marker-less tracking; object interaction; audio-visive feedback; control-volume
**CONS**	No Entertaining; No quantities data; No feedback; No control-volume;	No patient-based; 2D world; No integrated data acquisition; No gesture recognition; No control-volume;	No entertaining; Motion sickness; No gesture recognition; Controller; No control-volume;	No Home-Care; Pricey; No patient-friendly; No patient-based; 2D world; No gesture recognition; No control-volume;	No entertaining; 2D world; No gesture recognition; No control-volume;	No patient-based; Pricey; Bulky; 2D world; No control-volume; No object interaction;	Pricey; No Home-Care;

## Data Availability

Not applicable.

## Supplementary Materials

The following supporting information can be downloaded at: https://www.mdpi.com/article/10.3390/s23031680/s1, Figure S1: Graphs of velocity and acceleration of the right hand registered in Exergaming 1. Figure S2: Graphs of velocity and acceleration of the left hand registered in Exergaming 1. Figure S3: Graphs of rotation ad angular velocity of the right hand registered in Exergaming 1. Figure S4: Graphs of rotation ad angular velocity of the right hand registered in Exergaming 1. Figure S5: Graphs of velocity and acceleration of the right hand registered in Exergaming 2. Figure S6: Graphs of velocity and acceleration of the left hand registered in Exergaming 2. Figure S7: Graphs of rotation and singular velocity of the right hand registered in Exergaming 2. Figure S8: Graphs of rotation and singular velocity of the left hand registered in Exergaming 2. Figure S9: Graphs of head angles of rotation registered in Exergaming 1 with the right hand. Figure S10: Graphs of head angles of rotation registered in Exergaming 1 with the left hand. Figure S11: Graphs of head angles of rotation registered in Exergaming 2 with the right hand. Figure S12: Graphs of head angles of rotation registered in Exergaming 2 with the left hand. Figure S13: Graphs of angles of rotation of the gaze direction registered in Exergaming 1 with the right hand. Figure S14: Graphs of angles of rotation of the gaze direction registered in Exergaming 1 with the left hand. Figure S15: Graphs of angles of rotation of the gaze direction registered in Exergaming 2 with the right hand. Figure S16: Graphs of angles of rotation of the gaze direction registered in Exergaming 2 with the left hand.
