# Supplementary material for: A Proof of Concept Combined Using Mixed Reality for Personalized Neurorehabilitation of Cerebellar Ataxic Patients
Source: Sensors (Basel). 2023 Feb 3;23(3):1680. doi: 10.3390/s23031680 (PMC9920853; doi:10.3390/s23031680)
Supplement: Supplementary file 1 [file sensors-23-01680-s001.zip › sensors-2146265-supplementary.pdf]

SUPPLEMENTARY MATERIALS

The graphs are divided by exergaming and show information on the quantities measured by the HoloLens2. Speed and acceleration (Figure S1, S2, S5 and S6), angles and angular velocity (Figures S3, S4, S7 and S8) of the hands are obtained from the device's tracking algorithm. Head angles of rotation (Figures S9, S10, S11 and S12) are measured by the IMU sensor. The angles of rotation of the gaze direction (Figures S13, S14, S15 and S16) are obtained by a proprietary algorithm. HoloLens2 shows sufficient accuracy in following the movement and acquiring data for a rehabilitative application, although the detection of the gaze direction could be improved.

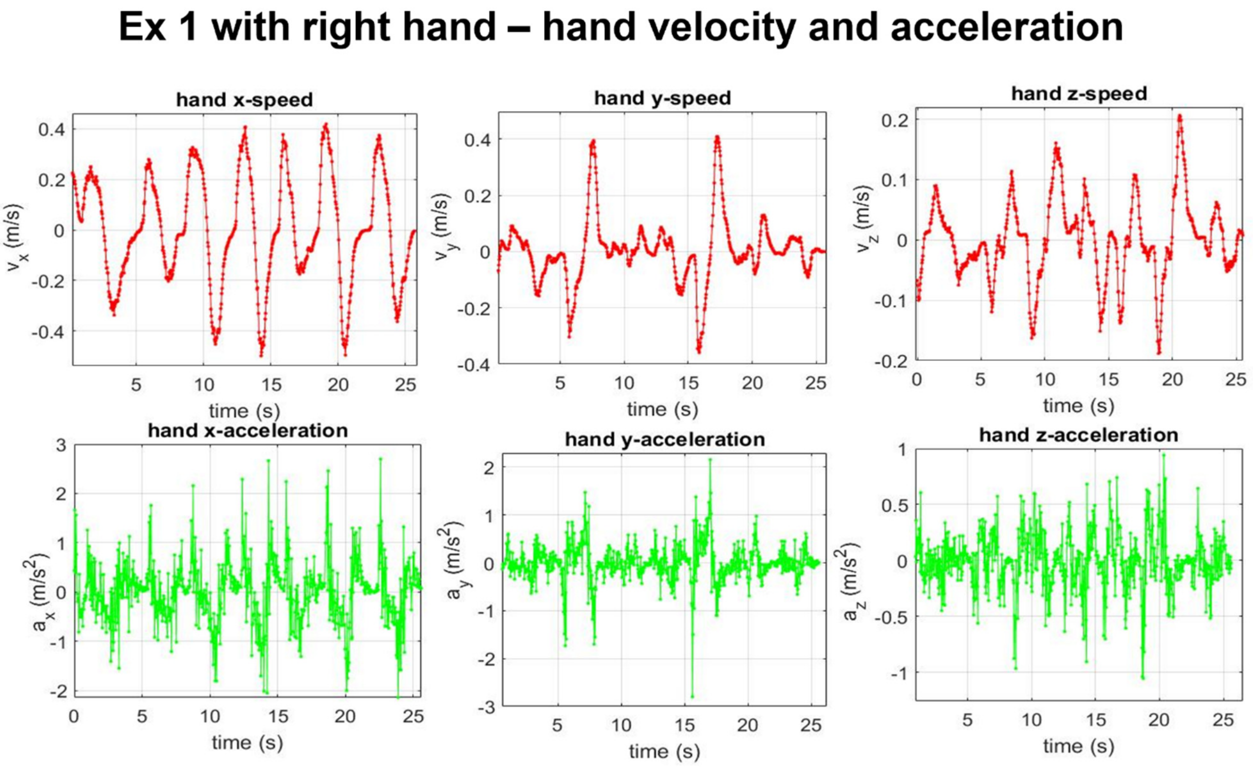

Figure S1 Graphs of velocity and acceleration of the right hand registered in Exergaming 1.

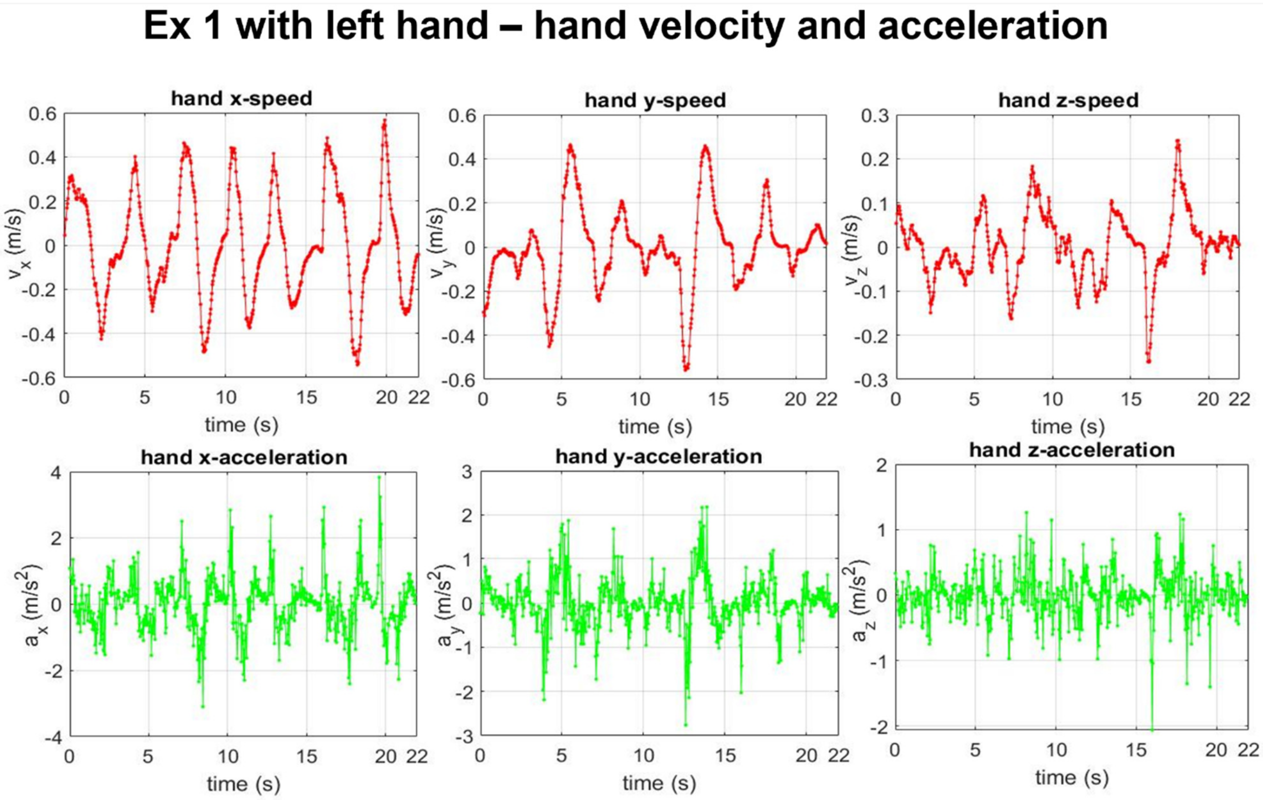

Figure S2. Graphs of velocity and acceleration of the left hand registered in Exergaming 1.

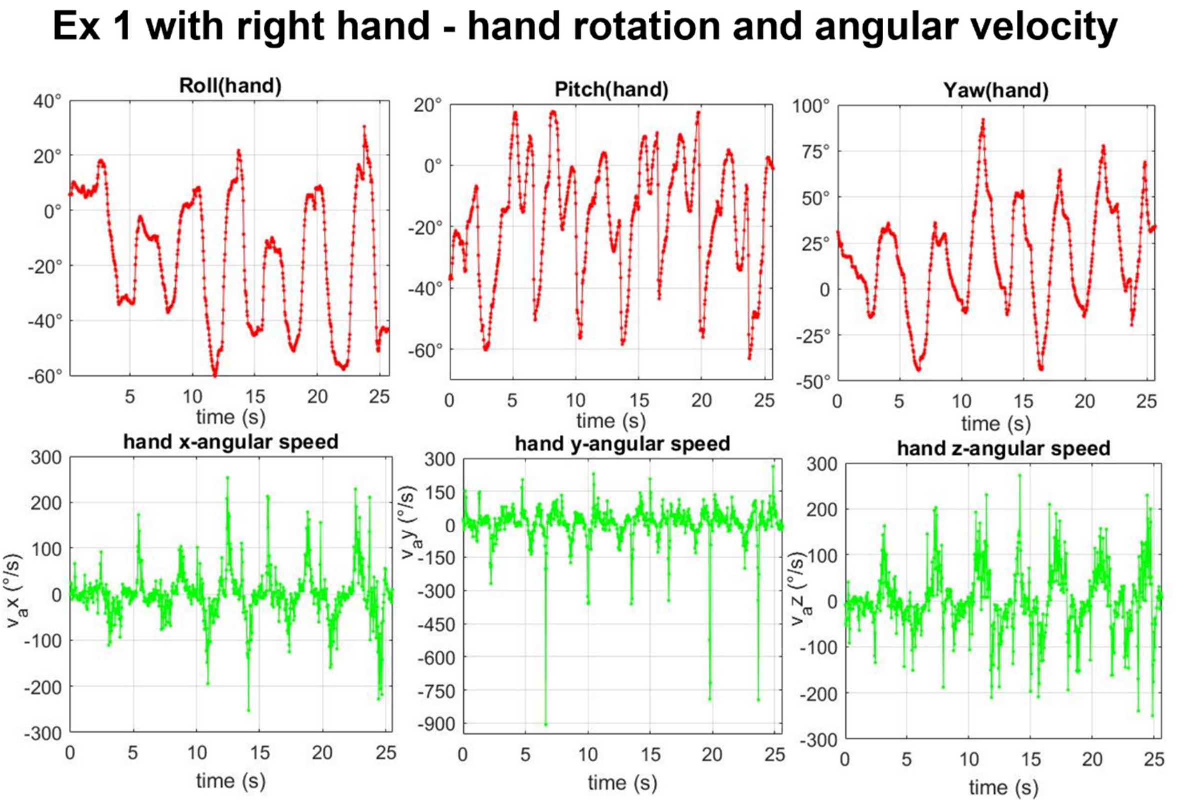

Figure S3 Graphs of rotation ad angular velocity of the right hand registered in Exergaming 1.

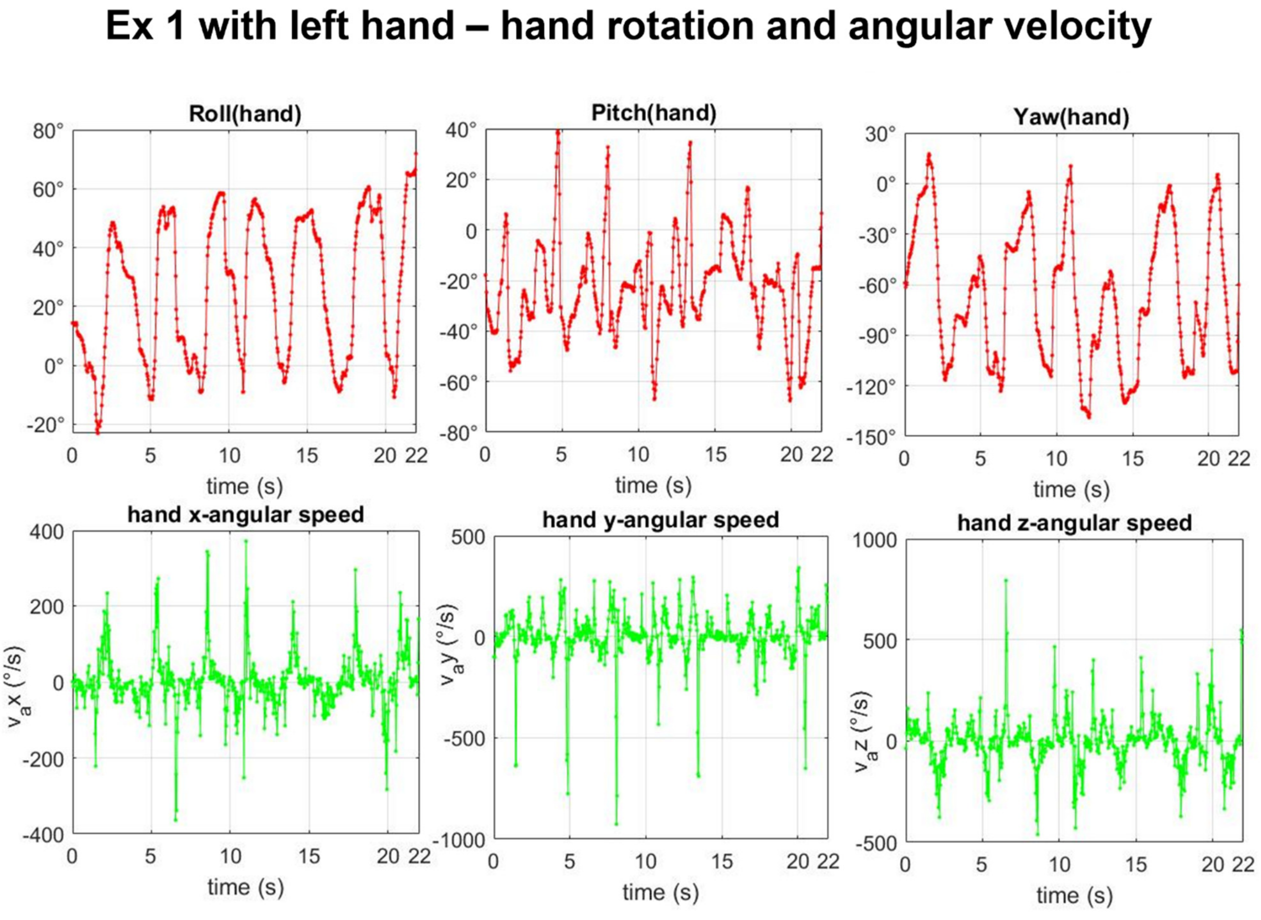

Figure S4. Graphs of rotation ad angular velocity of the right hand registered in Exergaming 1.

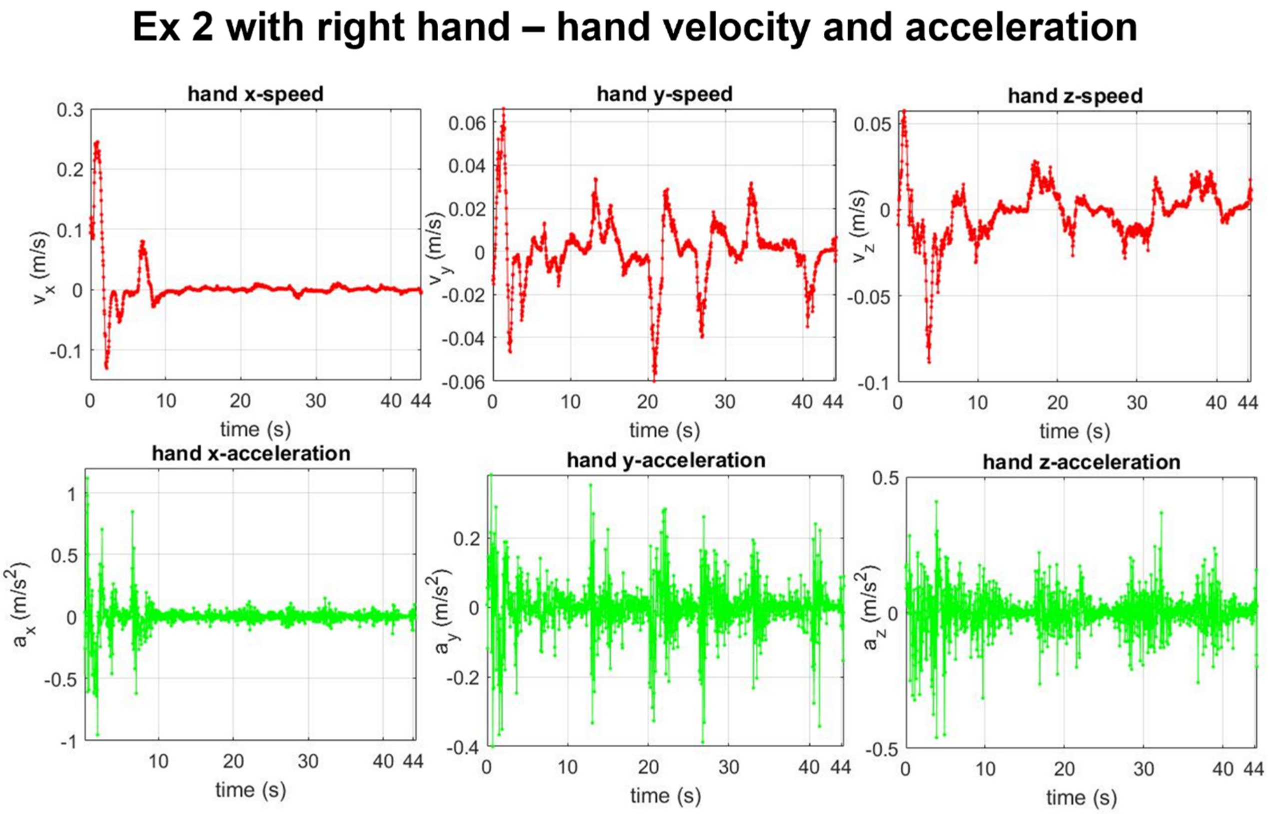

Figure S5. Graphs of velocity and acceleration of the right hand registered in Exergaming 2.

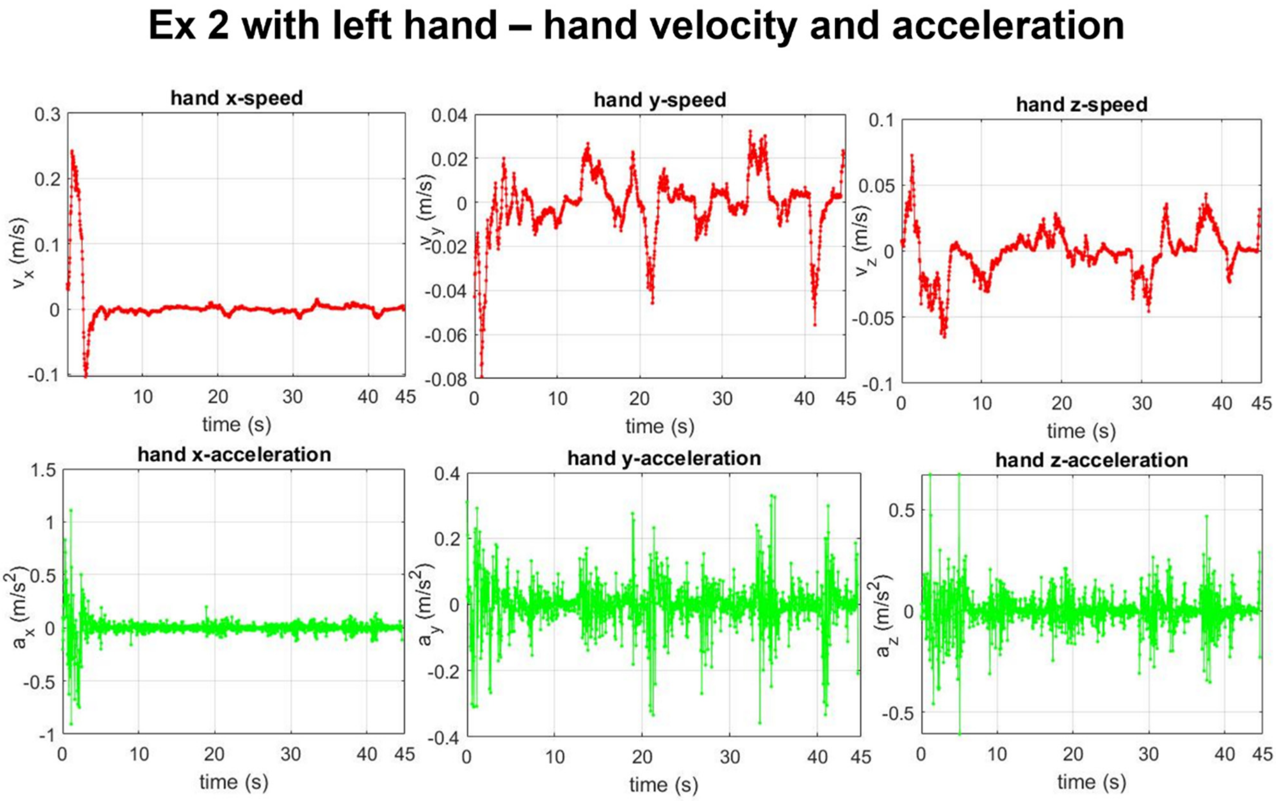

Figure S6. Graphs of velocity and acceleration of the left hand registered in Exergaming 2.

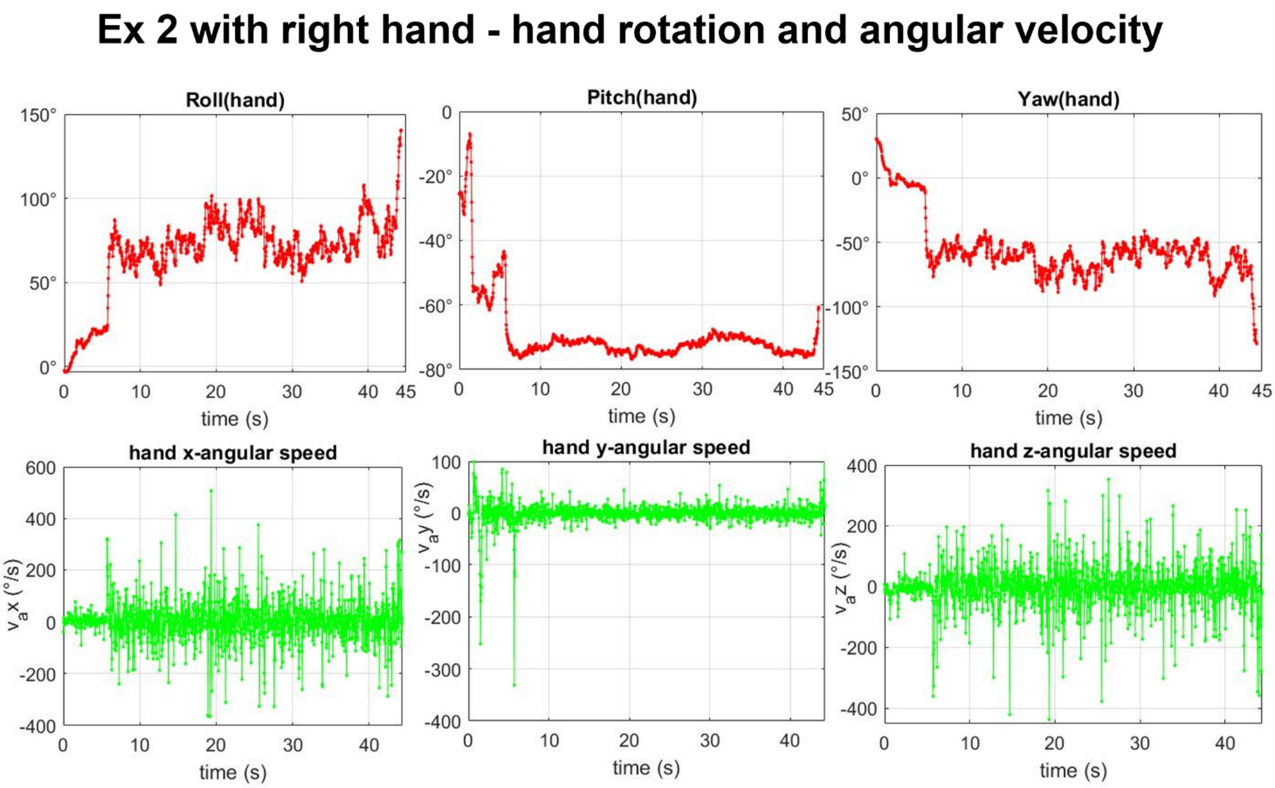

Figure S7. Graphs of rotation and singular velocity of the right hand registered in Exergaming 2.

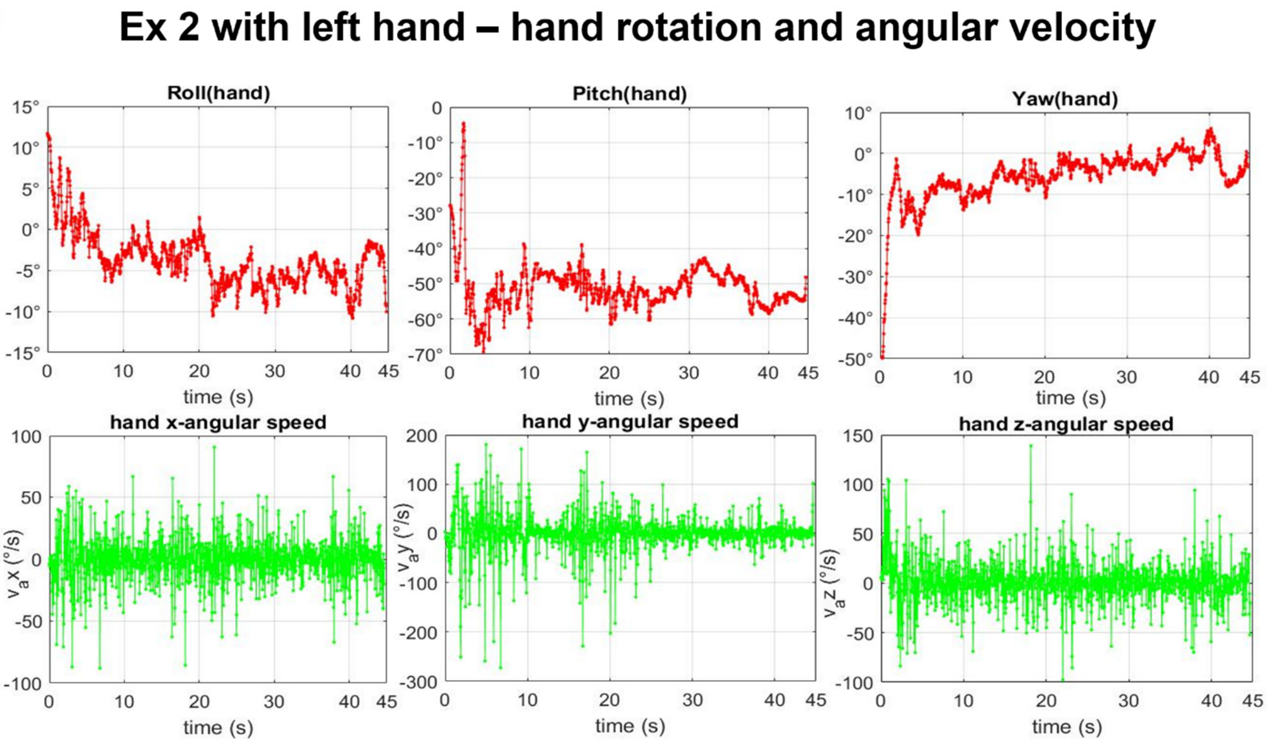

Figure S8. Graphs of rotation and singular velocity of the left hand registered in Exergaming 2.

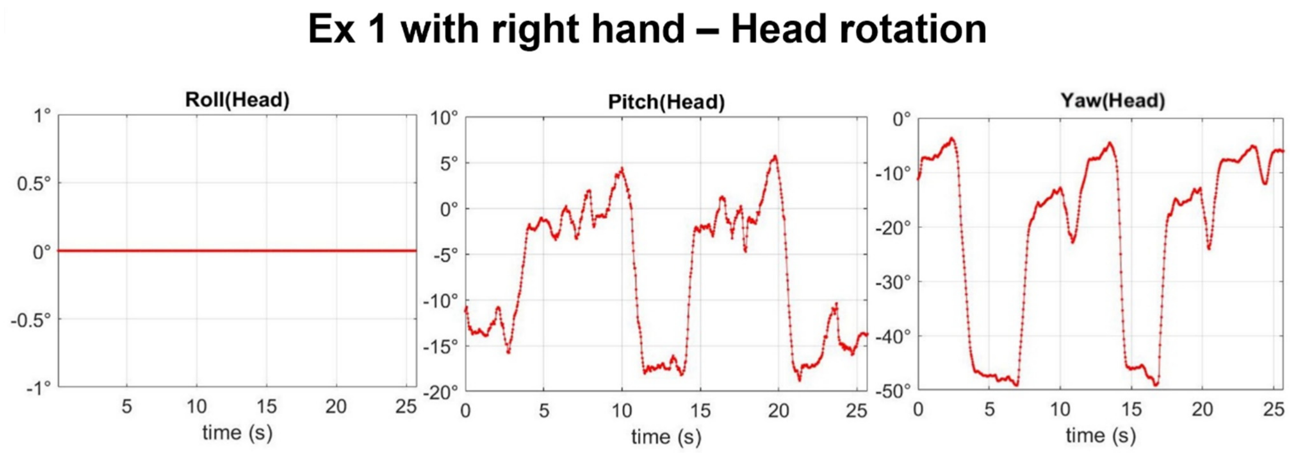

Figure S9. Graphs of head angles of rotation registered in Exergaming 1 with the right hand.

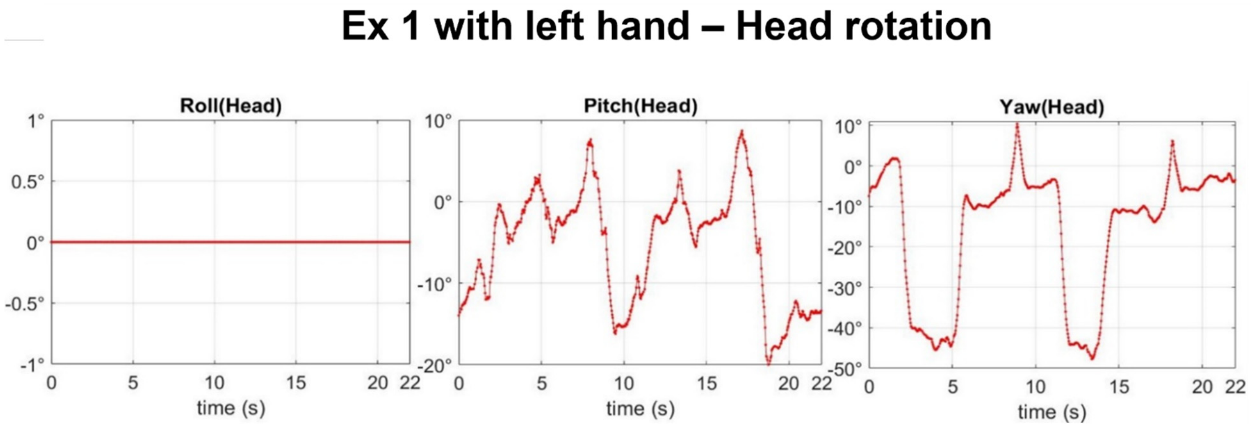

Figure S10. Graphs of head angles of rotation registered in Exergaming 1 with the left hand.

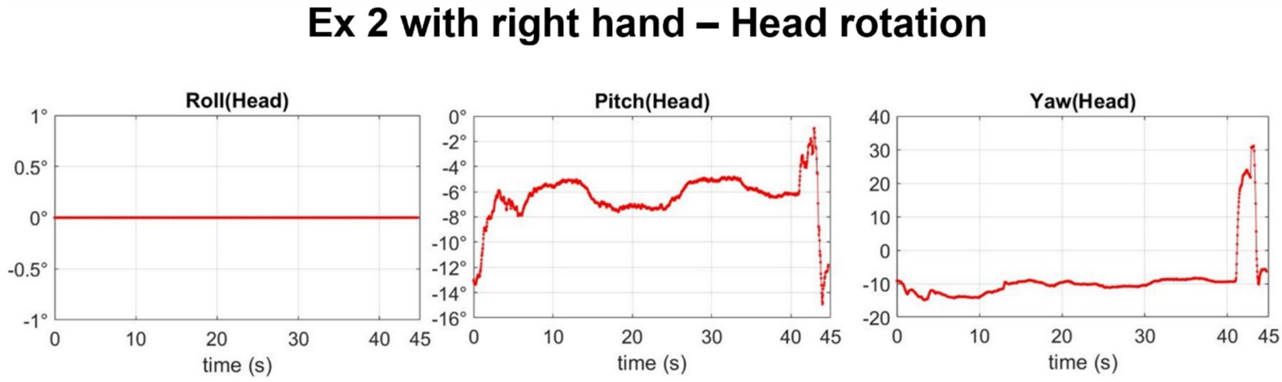

Figure S11. Graphs of head angles of rotation registered in Exergaming 2 with the right hand.

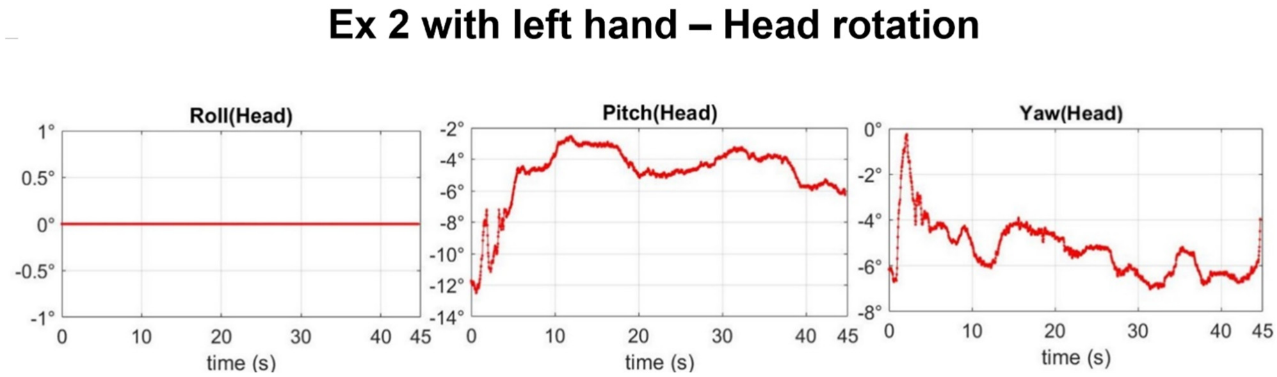

Figure S12. Graphs of head angles of rotation registered in Exergaming 2 with the left hand.

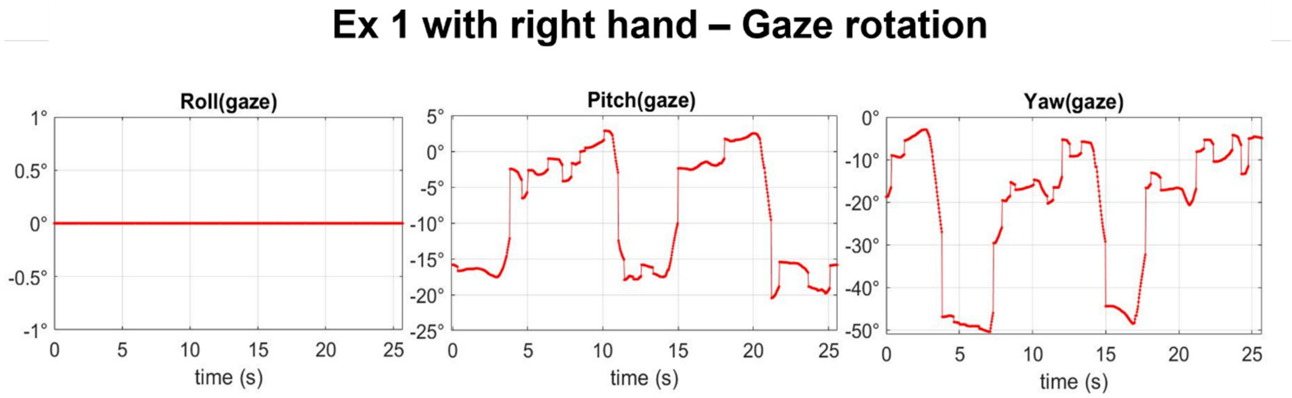

Figure S13. Graphs of angles of rotation of the gaze direction registered in Exergaming 1 with the right hand.

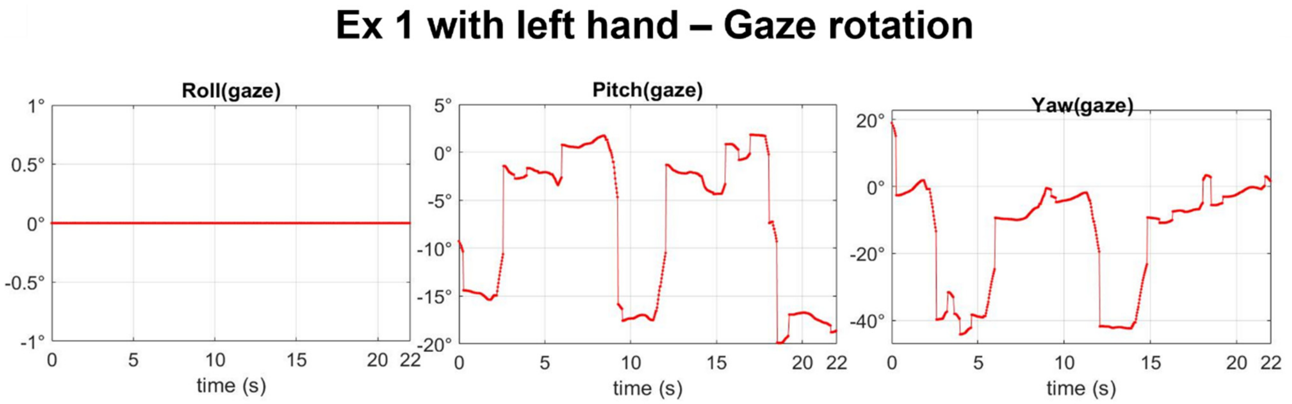

Figure S14. Graphs of angles of rotation of the gaze direction registered in Exergaming 1 with the left hand.

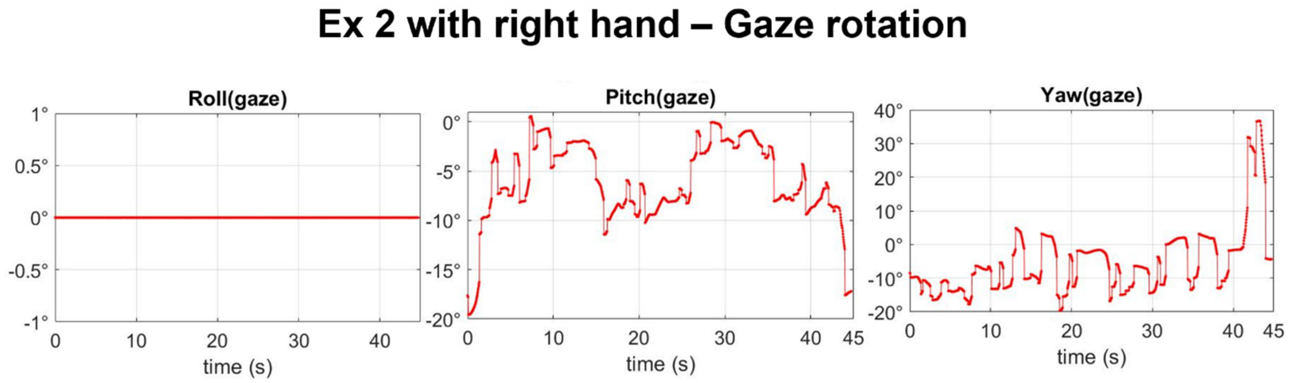

Figure S15. Graphs of angles of rotation of the gaze direction registered in Exergaming 2 with the right hand.

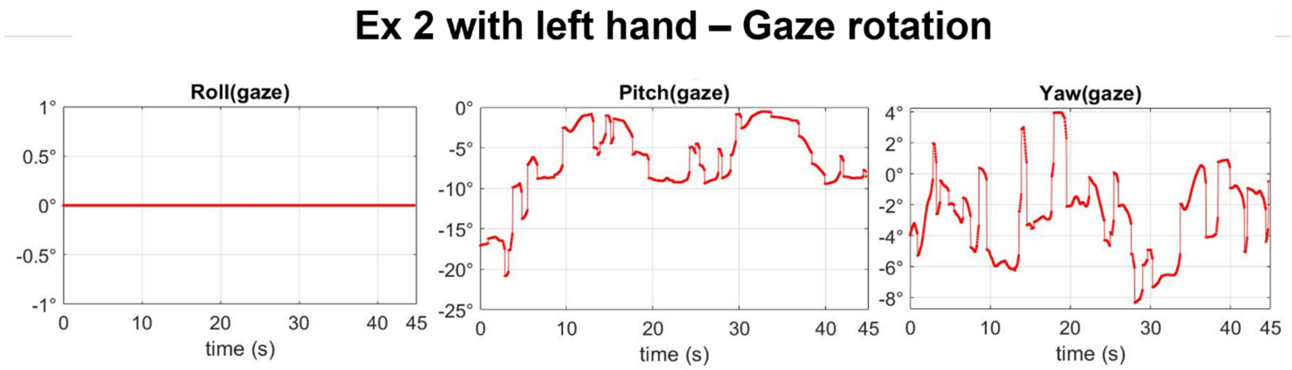

Figure S16. Graphs of angles of rotation of the gaze direction registered in Exergaming 2 with the left hand.
